# Factors that influence children’s gambling attitudes and consumption intentions: lessons for gambling harm prevention research, policies and advocacy strategies

**DOI:** 10.1186/s12954-017-0136-3

**Published:** 2017-02-17

**Authors:** Hannah Pitt, Samantha L. Thomas, Amy Bestman, Mike Daube, Jeffrey Derevensky

**Affiliations:** 1 Centre for Population Health Research, School of Health and Social Development, Faculty of Health, Deakin University, 221 Burwood Highway, Burwood, Victoria 3125 Australia; 2 Faculty of Health Sciences, Curtin University, Perth, Australia; 3 International Centre for Youth Gambling Problems and High Risk Behaviours, McGill University, Montreal, Canada

**Keywords:** Gambling, Marketing, Family, Media, Consumption, Children

## Abstract

**Background:**

Harmful gambling is a public health issue that affects not only adults but also children. With the development of a range of new gambling products, and the marketing for these products, children are potentially exposed to gambling more than ever before. While there have been many calls to develop strategies which protect children from harmful gambling products, very little is known about the factors that may influence children’s attitudes towards these products. This study aimed to explore children’s gambling attitudes and consumption intentions and the range of consumer socialisation factors that may influence these attitudes and behaviours.

**Methods:**

Children aged 8 to 16 years old (*n* = 48) were interviewed in Melbourne, Australia. A semi-structured interview format included activities with children and open-ended questions. We explored children’s perceptions of the popularity of different gambling products, their current engagement with gambling, and their future gambling consumption intentions. We used thematic analysis to explore children’s narratives with a focus on the range of socialising factors that may shape children’s gambling attitudes and perceptions.

**Results:**

Three key themes emerged from the data. First, children’s perceptions of the popularity of different products were shaped by what they had seen or heard about these products, whether through family activities, the media (and in particular marketing) of gambling products, and/or the alignment of gambling products with sport. Second, children’s gambling behaviours were influenced by family members and culturally valued events. Third, many children indicated consumption intentions towards sports betting. This was due to four key factors: (1) the alignment of gambling with culturally valued activities; (2) their perceived knowledge about sport; (3) the marketing and advertising of gambling products (and in particular sports betting); and (4) the influence of friends and family.

**Conclusions:**

This study indicates that there is a range of socialisation factors, particularly family and the media (predominantly via marketing), which may be positively shaping children’s gambling attitudes, behaviours and consumption intentions. There is a need for governments to develop effective policies and regulations to reduce children’s exposure to gambling products and ensure they are protected from the harms associated with gambling.

## Background

The impact of gambling on the health and wellbeing of individuals, families and communities has become an increasingly discussed and debated public health issue. With the advent of new technologies making gambling products and opportunities more accessible in our environments than ever before, governments are considering how best to respond to the potential risks and benefits posed by these potentially harmful products. While there has been significant and important evidence about the harms caused by some forms of land-based gambling, such as electronic gambling machines (EGMs, “pokies” or “slots”) [1–3], much less is known about the impacts of newer forms of gambling, such as online sports betting. This evidence gap is important given that many jurisdictions that have legalised online gambling are now playing “catch up” with regulatory frameworks seeking to prevent and minimise the harms associated with the provision and promotion of these products [4]. While many countries are currently considering the legalisation of sports and online betting [5], there is limited research evidence about the potential short- and long-term public health impacts of introducing these products, both on those who are legally allowed to gamble, and on children who are exposed to marketing for these products.

Australia provides an important case study for policy makers seeking to understand the impact of newer forms of gambling products on population subgroups [6]. Research suggests that excessive gambling may contribute to many different types of health and social harms, including financial harm, relationship conflict and breakdown, detriments to health, disruptions with study and/or work, cultural harm, and criminal activity [7]. Australians spend more money per capita on gambling than any other country in the world [8], with 2014/15 figures estimating that Australian adults spend on average $1241 per person on gambling each year [9]. While there have been decreases in participation in some forms of gambling, the largest increases in spend have been for online sports betting [9], and recent research estimates that about 11% of sports betting expenditure can be attributed to people who are classified as problem gamblers [10]. There have also been significant increases in advertising for some gambling products, with a 160% increase in advertising spend since 2011 [11]. Research has shown that sporting matches in particular have a high volume of marketing for gambling products [12–14]. This has stimulated considerable community debate about the impact of marketing on the normalisation of gambling for children, who make up a significant proportion of professional sport fans.

While most countries have a legal age for regulated forms of gambling (in Australia 18 years of age), evidence suggests that approximately two thirds to three quarters of children will have participated in some form of gambling in their pre-teen and teenage years [15–18]. While research shows that children mostly engage in “soft” forms of gambling such as lotteries or scratch cards, much of this research pre-dates the more pervasive and promoted online forms of gambling [17]. Similarly to adults, a broad range of harms are associated with children’s gambling behaviours, including mental health problems, issues associated with self-esteem and self-confidence, truancy, a reduction in academic performance, and other risk-taking behaviours [17, 19].

### Children and gambling: the role of consumer socialisation

Theories relating to consumer socialisation have been central to research that seeks to understand how and why children decide to consume products that may be harmful for them. Defined by Ward (1974) as “processes by which young people acquire skills, knowledge, and attitudes relevant to their functioning as consumers in the marketplace” [20] (pg. 2), socialising agents are factors that the “learner” interacts with and which are used to “transmit norms, attitudes, motivations and behaviours” [21] (pg. 600). They are traditionally associated with family, peers, and the media (including marketing) [21]. The impact and influence of these socialising agents can have a different effect on individuals depending on their life stage and individual make-up [20]. For example, in relation to marketing John (1999) has proposed that at different developmental stages, children start to develop different abilities and skills that they use in consumer decision making [22]. John (1999) argues that from the ages of about 3 to 7 years old, children are able to recognise brands, but have limited understanding of the persuasiveness of marketing and thus are unable to make informed consumer decisions [22]. Between the ages of about 7 and 11, children are able to understand the selling intentions of advertising, and purchasing and selecting products, but they still lack the skills to operate as sophisticated consumers in the marketplace. Finally, John (1999) states that older children (approximately 11- to 16-year-olds) are more reflective about consumer decisions and are able to build upon information to which they have previously been exposed but are also influenced by the opinions of others to make more informed consumer decisions [22]. While some would argue that parents may have some influence over children’s decision making, others argue that new media environments mean that after a certain age parents have limited influence in mediating children’s preferences for highly attractive products (for a summary see Calvert (2008) [23]).

A range of different socialisation factors may influence children’s gambling attitudes and consumption intentions. For example researchers have shown that socio-cultural factors, such as the influence of family members, and peers, may play important roles in facilitating children’s gambling behaviours [24, 25]. Researchers have also demonstrated that children’s first formal contacts with gambling are often via parents or family members [18, 25–28]. Children who believe that their parents gamble are more likely to want to try gambling themselves, and have higher rates of gambling [29]. Perceptions of the behaviours of peers may also influence young people’s attitudes and consumptions intentions towards gambling products [25, 30]. For example, research has demonstrated that peer-based gambling may also lead children (and in particular girls) to gamble more than they would if they were on their own [31]. The perceived popularity of products also plays an important role in children’s uptake of products [32]. However, there is very limited information about whether young people may perceive some types of gambling as being more popular, and perhaps more importantly what may influence these perceptions. While research has previously suggested that electronic forms of gambling are not particularly attractive for young people [33], these studies pre-date the newer and more pervasive forms of online gambling and the associated marketing for these products [34]. Furthermore, researchers have suggested that the emergence of gambling via digital media platforms may make gambling more “ubiquitous and socially acceptable” for children [35] (pg. 175). Finally researchers have investigated how gambling environments, and the promotion of gambling within these environments, may contribute to the normalisation of gambling in children. For example researchers argue that gambling may be normalised for children who attend gambling venues which are also promoted as “family friendly” [36] and that the alignment between gambling marketing and sport may have a significant influence on normalising gambling for young people [37, 38]. Research indicates that advertising may have an impact on children’s recall of and preference for gambling products [38], their attitudes towards gambling [24, 34, 38] and their perception that gambling is a normal or common part of sport [37].

Concerned about the impact of gambling advertising on children, politicians, policy makers, the media, academics and community members in Australia and the United Kingdom (countries with significant amounts of televised gambling advertising) have strongly advocated for prohibiting the promotion of gambling prior to the watershed (the time at which adult content can be shown on television) [34, 39, 40]. However, there is very limited knowledge about how marketing may interact with other socialising agents to positively shape children’s gambling attitudes, product preferences and consumption intentions.

The following study aimed to contribute to our understanding of how a range of consumer socialisation processes may shape children’s gambling attitudes and gambling consumption intentions. The study was guided by three broad research questions:

1. Are there specific socialisation factors that may positively influence children’s understanding and perceptions of the popularity of specific gambling products?
2. Do some factors appear to be more influential than others in shaping children’s gambling attitudes and consumption intentions?
3. How can public health strategies be used to reduce the harms associated with socialising agents which are particularly influential in positively shaping children’s gambling attitudes and consumption intentions?

## Methods

### Approach

The data presented in this paper was part of a broader study with parents and children investigating their attitudes and perceptions towards gambling. When developing this broader study, we utilised Constructivist Grounded Theory (CGT) methods in the development of research questions, and the collection and analysis of the data [41]. This is because we were interested in the social processes that may be influencing or shaping children’s gambling attitudes and perceptions. CGT also describes the dynamic role that both researchers and participants play in co-creating meaning about a particular topic or issue [41], and has been used in a number of different studies investigating gambling behaviours [24, 42, 43]. CGT principles were applied in a variety of ways throughout the study. For example, our interest in socialisation factors led us to theoretically sample family groups so that we could investigate the interaction between parents and children.

The data presented in this paper focuses only on information relating to children in the sample. It uses a thematic approach to the interpretation of the data, which aimed to identify conceptual patterns and links within and between children’s narratives specifically in relation to different gambling products. Ethical approval was received by the University Human Research Ethics Committee.

### Recruitment

Parents and children aged 8–16 years old were approached to participate in the study in Melbourne, Victoria, from April to July 2016. We chose this age group because research suggests that from about the age of 8 children start to understand the persuasive intent of marketing campaigns [22]. Given the particular focus of this study on gambling and sport, children had to play or be a fan of Melbourne’s major sporting code—the Australian Football League (AFL)—to be included in the study. We invited children’s participation in the study via their parents, initially using convenience-sampling techniques to approach parents with information about the study using local community networks (such as sporting clubs and community groups). A snowball sampling approach was subsequently used requesting parents of children who participated in the study to recommend other families who might be interested in participating. Finally, purposive sampling techniques were used to reach specific types of young people who might have had different experiences with or attitudes towards gambling products [44].

Parents were provided with an information sheet about the study and asked to discuss participation with their child or children. Two researchers attended the interviews at the family home with the lead author conducting most of the interviews with the children. Children were provided with information about the study prior to their participation and verbal consent was obtained. Multiple children from one family were allowed to participate as previous research has shown that children within family groups may hold very different attitudes towards different products [34]. At the conclusion of the study, the family received a $30 gift voucher for each participating child.

### Data collection

Face to face interviews were conducted with children using a semi-structured interview format. Interviews lasted between 25 and 45 min and were audiotaped with permission. In developing the interviewing techniques for this study, we considered in detail the potential power dynamics between the researchers and the children, as well as between parents and siblings, and how this could potentially influence children’s responses to the questions posed. We drew upon many of the processes described in other studies investigating the impact of gambling marketing on children [34, 37]. Children were interviewed away from parents and any other siblings. We utilised many “child-friendly” activities such as the use of picture boards at the start of the interview. We also thought extensively about the language that would be used when discussing gambling with children [45]. For example, previous research has shown that children are more likely to understand colloquial terms associated with gambling such as “betting” rather than the more formal term “wagering” [37].

In piloting the study, we also found that there was a degree of social desirability in children’s responses about gambling participation. In this context, we found that the framing of our questions was important in allowing children to expand upon their answers. Most children were aware that gambling was not allowed for children. For example, asking children if they wanted to “try sport betting” often elicited an immediate “no” response from younger children. However, if we followed this question with “what about when you are older, or when allowed to gamble?” young children were more open to discussing their gambling consumption intentions. It also provided us with an insight into the age at which children perceived that gambling was an acceptable activity. For example, some younger children described that they would engage in gambling as “teenagers” which they perceived was a more likely and “grown up” age for individuals to start to participate in gambling. We also noted that the structure of the interview was important. As such, we rearranged the order of questions for some children to introduce new concepts and to recall information that was discussed later in the interview [46].

Children were first asked general questions about themselves including their age and gender. This was followed by questions relating to children’s gambling behaviours. This included whether they had ever gambled before, which forms of gambling they believed were most popular, did they discuss gambling on sports with their family and friends, and which types of gambling, if any, would they like to try. A range of visual sociology techniques were incorporated throughout the interview as a creative way to stimulate discussion and to encourage children to think about questions in different ways [47]. Gambling is sometimes a complex issue for children to think about, and picture boards have been used in other studies to help children discuss their attitudes and opinions about different forms of gambling [37]. A number of interactive tools were used to prompt discussions about gambling. These included a picture board featuring pictures of eight forms of gambling—casino games, EGMs, horse racing, keno, lotteries, raffles, scratch cards, and sports betting. When speaking to children, colloquial language was used for some products, for example “scratchies” (scratch cards) and “poker machines” (EGMs). Children were then asked to circle the two forms of gambling they thought were the most popular (ranking their choices as first or second) and the activity they would like to try the most. Children were then asked qualitative questions about their choices.

### Data analysis

Interviews were transcribed by a professional transcription company, with QSR NVivo 10 being used to manage the data. Data were analysed throughout the interviews, starting from the first interview. This was used to adjust the interview schedule and also to guide our sampling strategies. We stopped collecting data and finalised the analysis when all aspects of the data were able to illustrate a number of concepts, and could be categorised in a way that was clear and able to answer the research aims [44].

The first author led the data analysis process, reading the interviews in their entirety, and then within family groups. Qualitative notes were regularly taken throughout the analysis process, with the first two authors meeting regularly to discuss the concepts emerging from the data. As each interview was completed, a process of coding occurred, with the researchers initially identifying broad codes, revising these to more specific codes as the data analysis progressed. Narratives were read several times and the meaning associated with children’s responses was constantly discussed. Where we were uncertain about the interpretation, we sought advice from the other researchers, who provided feedback until an agreed interpretation was reached. Where appropriate, we inserted tables to represent the key categories that had emerged from the data, and how these linked with different attitudes towards different products or different influences on behaviour. This is presented in the “Results” section of the paper (Table 2).

## Results

### General and gambling characteristics

The general and gambling characteristics of the sample are presented in Table 1. We interviewed 48 children from 30 family groups. The majority of children were male (*n* = 41; 85.4%), with just over half of children aged 12–16 years (*n* = 25; 52.1%). When we asked children about their participation in gambling, we did not distinguish between formal or informal gambling. Rather, we asked whether children had ever gambled before and then asked them to describe what they had participated in. Just under 40% (*n* = 19, 39.6%) of children described having engaged in either formal (using money to place bets on organised events, usually through family members) or informal gambling (demonstrated gambling knowledge and behaviours through creating situations where a valued object was wagered for something positive in return). Children were asked about their current and future intentions to gamble. A third of children indicated no desire to gamble currently or in the future (*n* = 16, 33.3%), over a third said they would like to gamble in the future but did not indicate they would like to try gambling currently (*n* = 18, 37.5%), a quarter (*n* = 12, 25%) said that they would like to try gambling now and also when they were older, and two children (4.2%) said they wanted to try gambling now but did not have any intention of gambling in the future.

**Table 1:** Children’s general and gambling characteristics

Gender	
Male	41 (85.4%)
Female	7 (14.6%)
Age	
8–11 years	23 (47.9%)
12–16 years	25 (52.1%)
Most popular product^a^	
Sports betting	23 (47.9%)
Lotteries	22 (45.8%)
Horse racing	21 (43.8%)
EGMs	13 (27.1%)
Casino games	5 (10.4%)
Keno	5 (10.4%)
Scratch cards	4 (8.3%)
Raffle	2 (4.1%)
Gambling product children would like to try	
Sports betting	17 (35.4%)
Lotteries	6 (12.5%)
Horse racing	6 (12.5%)
Casino games	6 (12.5%)
Scratch cards	5 (10.4%)
Raffles	3 (6.3%)
EGMs	1 (2.1%)
Keno	1 (2.1%)
No response	3 (6.3%)
Ever gambled	
No	29 (60.4%)
Yes	19 (39.6%)
Consumption intentions	
Desire to gamble in the future	18 (37.5%)
No desire to gamble	16 (33.3%)
Desire to try gambling now and in the future	12 (25.0%)
Desire to try gambling now but not in the future	2 (4.2%)

^a^Children could select two gambling activities as the most popular. The percentages reflect the number of children in the sample and not the number of choices

Three key qualitative themes emerged from the data.

### Factors that influenced children’s perceptions of the popularity of different gambling products

The first theme explores children’s perceptions of the popularity of different gambling products and the factors that they believed contribute to this popularity. A summary of the main factors can be found in Table 2.

**Table 2:** Factors that influenced children’s perceptions of the popularity of different gambling products

Sports and horse race betting
Game of “skill” not luck. Prolifically marketed on television. Aligned with culturally valued events.
Lotteries and scratch cards
Chance of winning lots of money. Perceived as less risky or “softer” forms of gambling. Small amount of money required to enter. Different lotteries available to enter.
EGMs and Keno
Children had seen the products before. Children had negative views of the risks and financial losses.
Casino games
Adult entertainment. Children had seen casinos in movies. A specific place to gamble.

Sports betting, lotteries and horse race betting were the three forms of gambling that children perceived were the most popular forms of gambling. Children had similar reasons for the popularity of sports and horse race betting. First was that sports betting, unlike other forms of gambling, was based on “skill” rather than “luck”. For example, some children described that people would bet on sports because they know about the teams and “know they are going to win”, with one 13-year-old commenting:

Well, I think if you watch sport more, you’re more likely to be able to guess what team is going to win because you could know which team is in better form.—13-year-old boy

Second, children believed that sports and horse race betting were popular because they were prolifically marketed on television. Children commented on the amount of marketing that they had seen for these activities, including that they had seen “a lot of ads for sports betting”, “heaps and heaps of ads for it like everywhere”, “it’s always on TV*”* and that “the majority of betting ads are horse racing ads”. Third, children commented that these forms of gambling were aligned with culturally valued events (such as sporting matches, and the Melbourne Cup racing event). For example, some children commented that sports betting would be popular because “sport is on all the time” and that “lots of people watch sport”. Other children described that horse racing events, and in particular the Melbourne Cup, were popular in Melbourne, and meant “lots of people bet”. Children often had an exaggerated perception of the popularity of formal betting on the Melbourne Cup, with one child stating that “millions of people do it”, and another child commenting that “everyone kind of bets on that [the Melbourne Cup]”.

Different factors influenced children’s perceptions of the popularity of other forms of gambling. For example, children perceived that lotteries and scratch cards were popular because there was a chance of winning a lot of money on these forms of gambling. For example, one child described that with scratch cards people had a chance of winning “something even if it isn’t much money”. Children also rationalised that lotteries and scratch cards were popular forms of gambling because they were less risky as compared to other types of gambling. This was mostly because children perceived that only a small amount of money was needed to play. Children also considered that there were “a lot of different lotteries” to play which would increase the popularity of the product. The following child believed the chance of winning was enough incentive to make people want to enter lotteries:

Well I know a lot of people consider lotteries as like, they don’t really consider it as full on gambling. But they do it just because, the chances are not really in their favour but they do it because…the slim chance of winning that amount of money is just enough for them—13-year-old boy

Those children who perceived that EGMs and Keno were popular chose these forms of gambling because they had seen them when having family meals at local pubs or clubs. Some children recalled seeing EGMs on specific occasions such as at “their beach house” and “through the window”. However, unlike other types of gambling, even when children chose EGMs as being a popular activity, they had a very negative view of the risks and financial losses associated with these games. Some children who thought that EGMs were popular also recalled that they were harmful for communities because of media attention relating to these machines:

There’s been a lot of talk about pokies recently on the news. And they rake in so much money each year. That’s why it’s such a big deal about getting rid of it.—15-year-old boy

One 8-year-old boy thought that EGMs were popular because they required people to continue to put money into them:

Because I know with pokie machines, you put money into them and then if you lose you have to keep on putting money in – until you’re poor.—8-year-old boy

Finally, a small number of children perceived that casino games were popular because they were considered as adult forms of entertainment. For example, a few children described that they thought adults “enjoyed a night out at the casino”, that they had seen casino gambling in movies, and that casinos were a specific place where people went to gamble.

### Factors that influence children’s gambling behaviours

Nineteen children in this study described that they had engaged in gambling (either formally or informally). Two main factors influenced young people’s gambling consumption behaviours. The first was the influence of family members and other adults in participating in gambling, and the second was the link between gambling and culturally valued events. These two factors were often intertwined. While a few children specifically reported having gambled on scratch cards and on Keno, most children who had participated in gambling had bet money on horse races or sporting events: “I’ve done horse racing with one or two dollars”. Children’s gambling was mostly linked to betting with or against adults. Sometimes children described engaging in “fun” bets with family members and family friends. While these bets rarely involved money, they related to specific events during sporting matches, such as which player would kick the most goals. The following child described how he placed bets with a family friend, and with his grandmother, about specific outcomes associated with matches. The child emphasised that he had won the bets, and that the person he was betting against was expected to follow through with their agreement:

I bet my Dad’s friend 10 push ups if Geelong would beat the Western Bulldogs [AFL teams]. I won. I also bet my Nana 10 push ups that, Tom Hawkins or Daniel Menzel [Geelong Cats football players] would score the first score and Daniel Menzel did, so 10 push ups.—9-year-old boy

Most children bet with either their own pocket money, or money given to them by their parents. Children who had participated in betting on the Melbourne Cup horse race rarely perceived that they had been involved in gambling. For example, the following child stated that he had never gambled but had used his pocket money in a sweep for the Melbourne Cup:

No [I haven’t gambled]. Well, for the Melbourne Cup, we did a sweep, where I paid just like $5 or $10 of my pocket money*.*—10-year-old boy

Some children described that betting on the Melbourne Cup was an exception from gambling, because other than this event they had otherwise never participated in gambling.

Well, once my Dad let me put $10 on the Melbourne Cup but other than that, no [I haven’t gambled]—13-year-old boy

While some children gave examples of gambling with their parents or other family members, particularly during the Melbourne Cup horse race, they rarely conceptualised this as a “real” form of gambling and often reported never discussing gambling with their family. For example, some children stated that they did not really talk about gambling with their family unless “it’s the Melbourne Cup”, when they discussed “who we think is going to win” and how they were going to place bets on different horses. One child described how they picked horses: “we usually do it off their names and like the random kooky names.” Some children also described entering sweeps with their family. Another 8-year-old boy described the Melbourne cup sweep as an annual family event:

So we get a newspaper and we cut up all the names of the horses and then we give out an even amount to everyone. I put on a bet, but my Mum did it for me.—8-year-old boy

### Factors contributing to current and future gambling consumption intentions

Finally, we explored the factors that influenced children’s reported future gambling consumption intentions. A third of children in this study indicated that they would never gamble. The main reason that children did not want to try gambling was related to a fear of losing money. This was mostly due to children remembering family discussions about gambling being a “waste of money”. Some children thought about adult-related scenarios such as needing to provide for a family when they were older, with one child describing that he would “get a job and make money, not try and win it that way.” These children also perceived that if they spent money on gambling they would not be able to afford other valuable items when they were older. For example one 8-year-old boy said he wanted to be able to spend his money on buying “a dog, house and car”.

Many other children described that they were curious about gambling and wanted to “see how it is” and “try it at least once”. However, other children were cautious about gambling, noting that it was something they would do “maybe a couple of times, but not often”, or would only gamble “just a few dollars”. Even when children said that they had discussed risks associated with gambling with their parents, some still indicated that they would like to try gambling “at least sometimes” when they were adults. Some clarified this by saying they would not gamble all the time, but would gamble only now and again “in case you get addicted”.

Four factors influenced young people’s current or future intentions to consume gambling products: (1) the alignment of gambling with culturally valued activities; (2) their perceived knowledge about sport; (3) the marketing and advertising of gambling products (and in particular sports betting) and (4) the influence of friends and family.

First, several children perceived that some forms of gambling, in particular sports and horse race betting, were a “normal” or culturally accepted activity. For example, one 11-year-old child stated that he would bet when “the bigger horse races are on”. Some children believed that it was almost compulsory for Australians to have a bet at least once on a major event:

It’s the kind of the thing you have to do at least once. Maybe something on a grand final [Football Match] or something.—14-year-old boy

Some children perceived that betting would make these events more fun and exciting, particularly if you were “winning some money.” For example, one 13-year-old boy said that he had thought about trying sports betting, but justified this response by saying that he “wouldn’t do it more than once or twice because then you might get addicted.”

Second, children who believed that they were knowledgeable about sports perceived that betting was an easy way to make money. These children believed that betting was a “skill” and that their knowledge about sporting events or teams indicated that they felt more confident about being able to pick winners by identifying “who is good and who is bad, who has the good defenders”. Children often stated that they would “probably bet on ‘my team’ sometimes” because it was the team that they knew the most about. A few children described that the most sensible time to bet was when there was a team or horse that would be a “clear winner” in a match or race. Children had a strong belief that knowledge of sports would positively influence the certainty of winning. For example, one 15-year-old girl explained that “if you have more knowledge about what team is better” you would be more likely to know who would win. The following 8-year-old also described the link between sporting knowledge both relating to teams and players and gambling success:

Well if you know a lot about the game you can usually pick the team that you reckon would win and then probably the best kick at goal.—8-year-old boy

Children who described very clear intentions to gamble when they were older described intricate scenarios where they would consider different betting options. Most of these scenarios involved AFL sporting matches. For example, the following 11-year-old using gambling language such as “punters” and “odds” described how he could use his understanding of gambling and the sporting form of two AFL teams to try to win more money. In this scenario, the child perceived that betting on the team with the longer odds and who was less likely to win would give him a chance of winning more money:

If it’s a clear winner or if it’s a really close game I might bet $10. Because I could get more money. And I would get more money, because I’d bet for a team that probably wasn’t going to win. If the odds were more, if the punters said Geelong [Geelong Cats AFL team] was going to win I’d probably go for Sydney [Sydney Swans AFL team] because it would be really close and they could win.—11-year-old boy

Third, children who had current or future gambling consumption intentions were strongly influenced by gambling advertising, particularly for sports betting. Children described that advertising made betting seem “easy” or “fun”, while others stated that gambling advertisements showed that “everyone wins”. Children described that advertising prompted them to actively think about trying gambling. For example, one 14-year-old boy described that he thought about trying gambling “when the ads constantly run”, telling the research team that he wanted to give sports betting “a crack”. Others stated that they thought about betting because of the incentives and promotions that were offered by betting companies. Children stated that taking up these incentives, in particular “cash back” or “refund” offers, would reduce their chance of losing money. For example, some children stated that they would gamble if there were promotions that offered “money back if your team is winning at half time but loses” or “if they say your team has good odds”. Incentive promotions were particularly influential in stimulating future consumption intentions for a few children who were unsure about whether they would gamble in the future. For example, a 10-year-old girl who was unsure about whether she would gamble when she was older said she would consider gambling if there was less risk involved. She went on to describe that “deals” promoted by bookmakers where she could get her money back if she lost, or would have a greater chance of winning a lot of money could encourage her to gamble:

Maybe if they had a deal or an ad and I think ‘oh I could get my money back if I do something or get heaps of money I might do it’.—10-year-old girl

Finally, a few children thought that friends and family members would influence their gambling when they were older. For example, a few children described that they thought “peer pressure” may play an influential role in gambling behaviours, or if it was normalised by “other people doing it around me.” Although family influences were a common theme that was influencing children’ s current gambling behaviours, it was not as present in children’s discussions of their future gambling consumption intentions.

## Discussion and implications for harm reduction initiatives

Before discussing the results from this study, it is important to highlight the study limitations. First, the sample was skewed towards boys and younger children and did not specifically seek to measure differences between children from different socio-demographic and ethnic backgrounds. This should be considered in future studies. This study recruited children who were fans of the AFL, which is a sporting code that has significant saturation of gambling marketing within its sporting matches [12]. For this reason, the children in this study may have had a heightened perception of sports betting compared to children who are fans of other sports which are not as heavily sponsored by gambling companies, or for children who are not fans of sport at all.

Table 3 suggests areas for future research, as well as strategies that may help to reduce the potential harms posed by these products to children.

**Table 3:** Suggestions for future research and harm reduction

Education campaigns
1. Education for children about the risks of gambling. 2. Education initiatives and public education campaigns for parents and children about the marketing strategies and tactics used by the gambling industry.
Regulation
1. Restricting gambling company advertisements from depicting gambling as a way of developing or building friendships and as a social activity. 2. Restricting gambling advertisements from sporting events and television broadcasts. 3. Restricting gambling advertisements that may have a high recall or appeal for young people.
Future research
1. Longitudinal research into children’s gambling consumption. 2. Research to investigate the age at which peers may start to become influential in gambling behaviours. 3. Research into newer gambling marketing techniques and the effect on children’s attitudes towards gambling.

Children in this study had much lower actual participation in both formal and informal gambling (about 40%) as compared to other studies. The lower rates of participation in this study as compared to other studies [15–17] could be due to the younger age of this sample or that children were asked to talk about their gambling behaviours in a face to face interview (rather than an anonymous survey). Nevertheless, the findings in this study suggest that children as young as 8 years old showed both current and future intentions to participate in gambling, in particular, sports betting. This may indicate that education about the risks of gambling should begin prior to adolescence and should aim to counter the overwhelmingly positive messages children see about gambling. There is also a role for education initiatives and public education campaigns, so long as these are developed independently of industry and part of a comprehensive public health approach, providing young people and their parents with clear information about the marketing strategies and tactics used by the gambling industry to promote their products. These campaigns could also challenge perceptions that some forms of gambling (such as sports betting) are based on “skill”. Research from other areas of public health, such as alcohol and tobacco, have demonstrated that the involvement of industry in the development of education-based campaigns is ineffective in reducing harm and may be counterproductive [48]. Some researchers have suggested that this is because these industries (and governments) are unwilling to implement education strategies that may ultimately impact on their profits (or taxation revenue) [49, 50] and may be a contributing factor in increasing children’s positive perceptions about these products [48, 51]. The implementation of gambling education initiatives may also play a further positive role in encouraging the community to demand more responsibility from sporting codes and broadcasters about their marketing relationships with the gambling industry, and more accountability from government to regulate how the gambling industry is able to promote their products.

Despite online sports betting being a relatively new form of gambling in Australia, nearly half of children chose this form of gambling as one of the two most popular types of gambling, and about a third stated that given a choice, they would try this form of gambling over other gambling activities. Popularity of products, and the early and repeat exposure to advertising, has been shown to have a significant influence on children’s long-term, and risky consumption behaviours of harmful products such as alcohol and tobacco [52–55]. While longitudinal research will provide evidence for gambling consumption over time, there is no reason to expect that the consumption trajectory for the heavily advertised sports betting would be any different to products such as alcohol or tobacco. It would therefore be appropriate for governments to adopt precautionary principals of harm reduction, with the burden of proof on the gambling industry to show that the marketing of their products will not influence risky patterns of gambling in young people either currently, or in the future, before they are allowed to expose young people to marketing for their products.

Children who had clear intentions to consume sports betting products believed that they would have a chance of winning because of their knowledge of the sport. Past research has found that children are more likely to experience harm from gambling because of their misunderstanding of perceived skill in chance-based games [56–58]. In this study, children clearly perceived that sports betting and to a certain extent horse race betting, were based on skill rather than chance. The regulations governments have implemented suggest they are conscious of children’s exposure to sports betting advertisements [59]. However, in play sports betting advertising is still currently allowed during sporting events. The most effective harm prevention and reduction strategies should involve government regulation to significantly reduce children’s exposure to advertising, particularly within sports.

Three primary socialising agents were influential in shaping children’s gambling attitudes and consumption intentions: family members, and in particular parents, culturally valued events, and marketing. Before discussing these, it is important to understand the factors that did not appear to have an influence on children’s behaviours. Unlike other areas of public health, such as alcohol and tobacco [60–62], and in other gambling studies [26, 31, 57, 63], peers did not appear to play a significant role in influencing the gambling attitudes and consumption intentions of this group of children. Further research should investigate the age at which peers may start to become influential in gambling behaviours, particularly given that many recent campaigns for betting companies are dominated by concepts of mateship [64]. Research has indicated that there is a process of symbolic consumption with these marketing strategies, with young men’s peer-based gambling behaviours reflective of the themes within sports betting advertising [65]. Further, there is research that has reported that sports betting in particular is being used as a form of social and group cohesion amongst groups of young male sports fans [65]. While further research is needed into the impact of these newer marketing creatives on young people, one harm reduction strategy may be to prohibit gambling companies from promoting gambling as an activity that helps to build peer relationships, or is a natural addition or complement to social activities.

The factor that appeared to have the most influence on young people’s current and future gambling attitudes and intentions to gamble was marketing for sports betting. Research from other areas of public health such as alcohol has shown that marketing which reinforces alcohol as a fun, social activity is likely to reinforce children’s normative assumptions about drinking [66, 67]. As has been demonstrated in other gambling studies [34, 68, 69], marketing of gambling as a socially acceptable behaviour has created a perception that gambling was “easy” and “fun” and that sports betting was different from other forms of gambling because it was based on skill. In addition, the research has shown that specific forms of marketing, such as inducements, may impact on children’s gambling attitudes and consumption intentions, particularly for children who were unsure about whether they would gamble when they were older. Further, even though many children had never gambled on a sporting event, they were able to describe different gambling markets, betting options, and “deals”. While the sports betting industry argues that the marketing for their products does not target children [11], children are nevertheless exposed to and influenced by the marketing messages that they see. Although we would expect that adolescents would be influenced and receptive to these messages, it is concerning that very young children also appear to be influenced by messages which are increasingly aligned to activities that are popular with children, such as sport. Prohibiting marketing for gambling prior to the watershed is important in limiting young people’s exposure to marketing; however, we would argue that comprehensive harm reduction approaches must go further. This includes regulating marketing strategies, including those outside of traditional television advertising, that have high recall or appeal for young people.

## Conclusions

This research suggests that a range of socialisation factors may be positively shaping children’s attitudes towards gambling products. As with other key areas of public health, a comprehensive approach to preventing the harms associated with gambling products will include a range of education and legislative responses. Given the new pervasive forms of gambling products, and the marketing for these products, government responsibility for the development of effective policies and regulatory structures will be critical in ensuring that young people are not exposed to gambling products and promotions in their everyday environments. Researchers will play a key role in mapping and monitoring industry tactics and their impact on children and using research evidence to advocate for change.

## Abbreviations

AFL: Australian Football League
CGT: Constructivist Grounded Theory
EGMs: Electronic Gambling Machines
